# Lateral prefrontal activity as a compensatory strategy for deficits of cortical processing in Attention Deficit Hyperactivity Disorder

**DOI:** 10.1038/s41598-017-07681-z

**Published:** 2017-08-03

**Authors:** Francisco Zamorano, Pablo Billeke, Leonie Kausel, Josefina Larrain, Ximena Stecher, Jose M. Hurtado, Vladimir López, Ximena Carrasco, Francisco Aboitiz

**Affiliations:** 1 División de Neurociencia, Centro de Investigación en Complejidad Social (neuroCICS), Facultad de Gobierno, Universidad del Desarrollo, Santiago, Chile; 2 Unidad de Imágenes Cuantitativas Avanzadas, Departamento de Imágenes, Clínica Alemana de Santiago, Santiago, Chile; 3 Centro Interdisciplinario de Neurociencias, Pontificia Universidad Católica de Chile, Santiago, Chile; 4 Escuela de Psicología, Pontificia Universidad Católica de Chile, Santiago, Chile; 5 Departamento de Psiquiatría, Escuela de Medicina, Pontificia Universidad Católica de Chile, Santiago, Chile; 6 Servicio de Neurología, Facultad de Medicina, Clínica Alemana Universidad del Desarrollo, Santiago, Chile; 7 Servicio de Neurología y Psiquiatría, Hospital Luis Calvo Mackenna, Facultad de Medicina, Universidad de Chile, Santiago, Chile; 8 Departamento de Imágenes, Facultad de Medicina, Clinica Alemana Universidad del Desarrollo, Santiago, Chile

## Abstract

Attention Deficit Hyperactivity Disorder (ADHD) is the most common neuropsychiatric disorder in childhood and is characterized by a delay of cortical maturation in frontal regions. In order to investigate interference control, which is a key function of frontal areas, a functional MRI study was conducted on 17 ADHD boys and 17 typically developing (TD) boys, while solving the multi source interference task (MSIT). This task consists of two conditions, a “congruent condition” and an “incongruent condition”. The latter requires to inhibit information that interferes with task-relevant stimuli. Behavioral results showed that ADHD subjects committed more errors than TD children. In addition, TD children presented a larger MSIT effect -a greater difference in reaction times between the incongruent and the congruent conditions- than ADHD children. Associated to the MSIT effect, neuroimaging results showed a significant enhancement in the activation of the right lateral prefrontal cortex (rlPFC) in ADHD than in TD subjects. Finally, ADHD subjects presented greater functional connectivity between rlPFC and bilateral orbitofrontal cortex than the TD group. This difference in connectivity correlated with worse performance in both groups. Our results could reflect a compensatory strategy of ADHD children resulting from their effort to maintain an adequate performance during MSIT.

## Introduction

Attention Deficit Hyperactivity Disorder (ADHD) is the most common neuropsychiatric disorder in childhood^1^, affecting between 5%^2^ and 15%^3^ of the school-aged population. While the incidence of this condition is greater among children in pre-school and school age, the disorder can persist into adolescence and adulthood in 30% to 60% of the cases^4^. During childhood, one of the main consequences of the syndrome is that children show poor school performance. During adolescence and adulthood, problems such as decreased self-esteem, social isolation, increased risk of accidents and psychiatric comorbidity become manifest, which are important in the context of public health^5–7^. Currently, it is posited that a good therapeutic outcome depends largely on early and appropriate treatment^8^, but also on a correct diagnosis and management of comorbidities that are usually associated with ADHD and that are often much more disruptive than ADHD itself^7, 9–11^. Mainly due to the complexity of the clinical and biological features of this disorder, its diagnosis remains controversial^12^. In this context, it is increasingly necessary to study more closely the neurobiological mechanisms underlying ADHD.

In the last years, structural MRI studies have shown that ADHD children present a delay in their cortical maturation^13^. Brain areas such as the insula and striatum are affected^14, 15^, even though the alterations in the prefrontal cortex might be the most prominent ones^16, 17^. It has also been shown that the degree of maturation delay in the right prefrontal region correlates with the severity of adult inattention symptoms^18^. Already in 1997, Barkley proposed that ADHD is caused by abnormalities in the prefrontal cortex and its connections to the striatum, resulting in severe alterations in inhibitory behavior^19^. Behavioral inhibition, also called interference control, includes self-directed responses that result from competing events and responses^19–21^. Although interference control is a relevant cognitive process in development, little is known about its functional maturation^22^. For example, some studies have found an increase in dorsolateral prefrontal activity associated with higher interference control^23^, while others have found a decrease in this activity parallel to improvement in performance during development^24, 25^.

**Figure 1:**
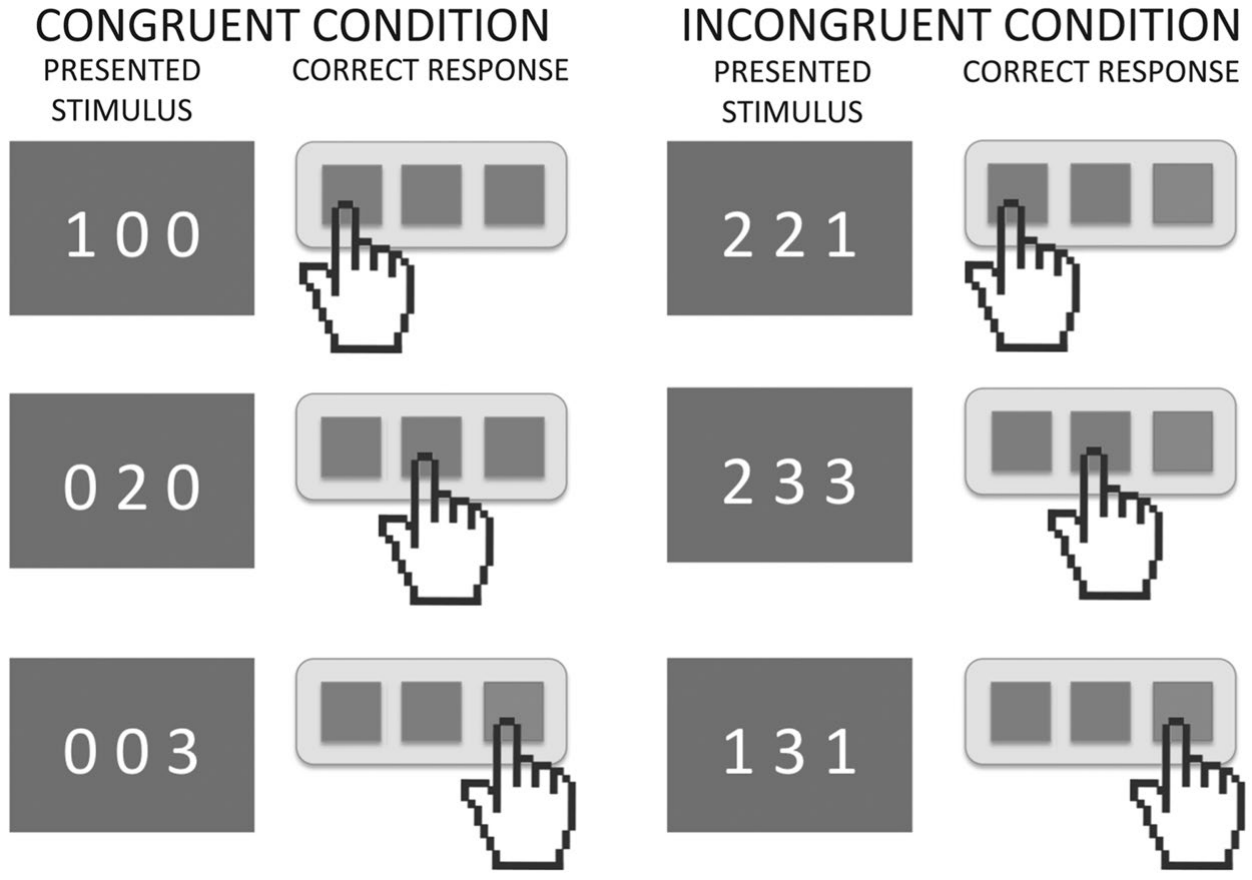
Schematic representation of the Multi Source Interference Task.

One of the most used cognitive paradigms to explore interference control is the Stroop Color-Word task^26–28^. This task has been paramount in the characterization of cognitive control and inhibition impairment in neuropsychiatry diseases^29^. Nonetheless, some authors indicated that other alterations (e.g., poor reading skills^30^) present in ADHD children may adversely affect the interpretation of the poor performance in this type of task^27^. In order to tackle this problem, recent studies have used the Multi-Source Interference Task (MSIT), which was designed as a block-formatted functional neuroimaging task and has shown to be effective to study response inhibition and attentional control^31^. Specifically, the MSIT presents 3 digits on a computer screen. Participants have to report the identity of the number that is different from the other two numbers by pressing a button on a response-pad. In the congruent condition (CC), the distracters are zeroes and the target number (1, 2, or 3) is always placed congruently with its position on the response-pad. In the incongruent condition (InC), the distracters are other non-zero numbers, and the target number (1, 2, or 3) is never placed congruently with its positions on the response-pad (Fig. 1). The contrast between conditions (InC > CC) robustly activates the cingulate fronto-parietal circuit network in adults and adolescents^24, 31^. Interestingly, adults with ADHD medicated for 6-weeks with either atomoxetine or methylphenidate show increased activity in right prefrontal and cingulate cortex during the MSIT task as compared to controls, even if no significant behavioral changes are observed^32, 33^. This evidence points to a dysfunction in inhibition processes as a relevant aspect of ADHD psychopathology^34^.

Since there is evidence for a maturation delay of the prefrontal cortex in ADHD children, this research tested the hypothesis that these children present an alteration in interference control that is related to the activity of prefrontal cortex. For this end we carried out a functional MRI study on 40 male children, 20 ADHD and 20 age and IQ-matched typical developing children (TD), while they were solving the MSIT (Table 1). Additionally, based on prior work indicating an increase in brain activity as part of a compensatory strategy in ADHD children, we expected that these subjects would present more errors and a greater activity in the cingulate fronto-parietal network as a trade-off to improve performance, as shown by Fassbender and Schweitzer^35^.

**Table 1:** Demographics of the participants.

	ADHD Mean (std. dev.)	CONTROL Mean (std. dev.)	Diff p-value
n	17	17	
Age (years)	11.6 (0.86)	11.7 (0.67)	0.87
IQ (Total)	104.2 (7.4)	109.8 (11.2)	0.084
Hyperactivity/Inattention Symptoms (Conners)	16.5 (4.9)	3.5 (4.1)	6.1e-5

## Results

### Behavioral results

Both groups demonstrated a significant accuracy decrease during the InC as compared to the CC (Mixed ANOVA, Condition factor: *F*_*4,29*_ = 29.8, *p* < 0.001). Even though the ADHD children committed more mistakes (Diagnosis factor: *F*_*30*_ = 4.2, *p* = 0.04), there was no interaction between condition and diagnosis (*F*_*4,29*_ = 2.0, *p* = 0.15). Considering only correct responses, both groups demonstrated equivalent significant increases in reaction time (RT) during InC as compared to CC (Mixed ANOVA, Condition factor: *F*_*4,29*_ = 11,4, *p* = 0.001, Diagnosis factor: *F*_*4,29*_ = 0.01, *p* = 0.97; Condition*Diagnosis: *F*_*4,29*_ = 0.17, *p* = 0.67). Interestingly, the MSIT-effect (individual contrast between InC – CC RTs), was greater in TD than in ADHD group (TD: 193.6ms, ADHD: 152.7ms, Wilcoxon test, w = 69, df = 33, *p* = 0.008).

**Figure 2:**
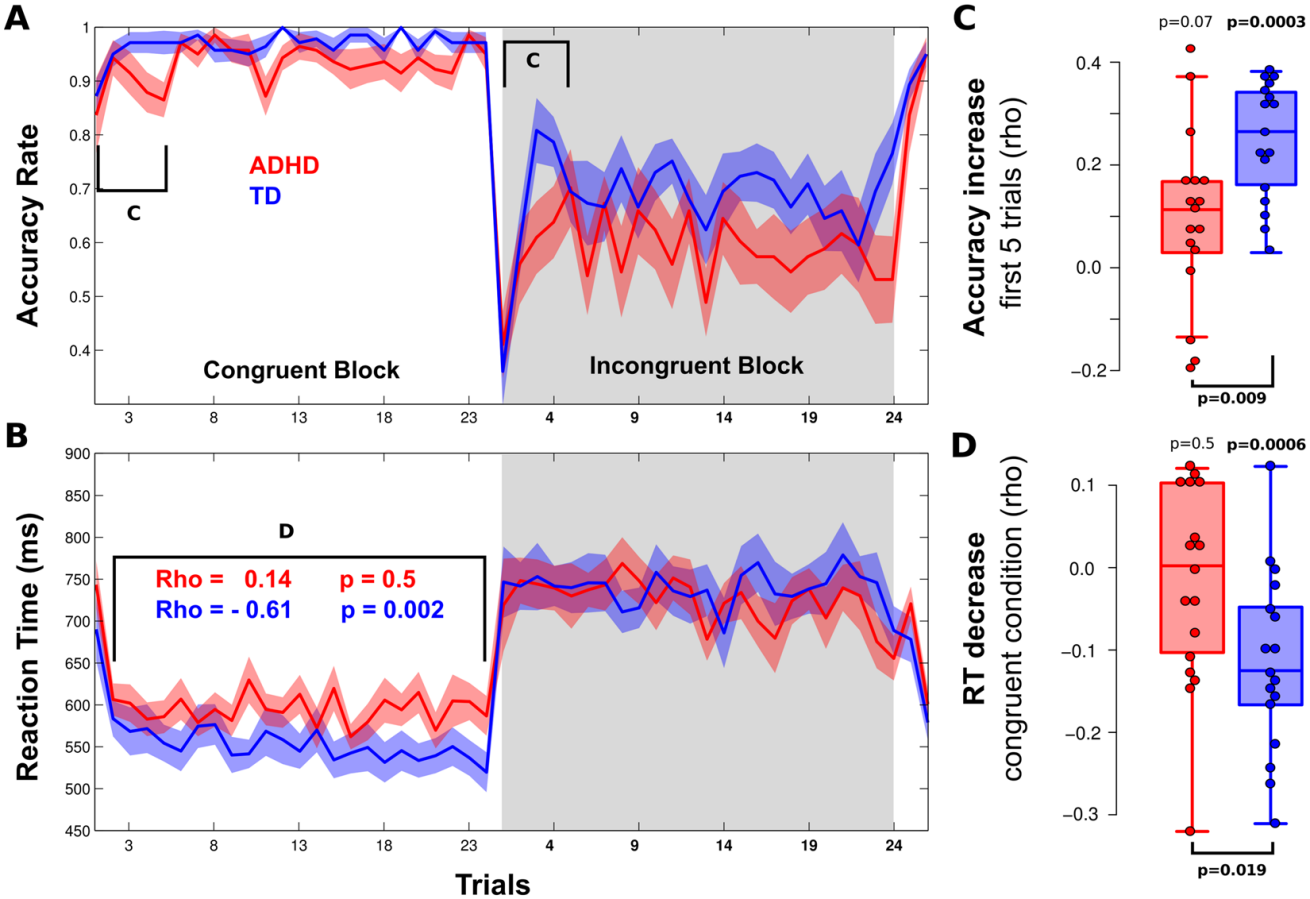
Behavioral Results. (**A** and **B**) Accuracy rate (**A**) and reaction time (**B**) in congruent (white background) and incongruent conditions (grey background) for ADHD (red) and TD (blue) children over the averaged block. Black brackets indicate trials considered for analysis in C and D respectively. (**C**) Accuracy increase over the first five trials of both conditions for ADHD (red) and TD (blue) children. (**D**) RT decreases over all congruent trials for ADHD (red) and TD (blue) children.

We next assessed for differences in the switching between CC and InC studying the performance dynamics during the task. For this, the accuracy of the five blocks per condition were averaged for each subject, obtaining one average block per subject. We observed that the increase in accuracy at the beginning of each condition block was poorer in ADHD than in TD subjects. Thus, we calculated the slope of this increase for each subject by means of a Spearman correlation between accuracy and trial number over the first five trials, pooling both conditions. The ADHD group demonstrated a lower correlation between accuracy and trial number than the control group (mean Rho ADHD: 0.07, *p = *0.07; TD: 0.25, *p* < 0.001; diff Wilcoxon test, w = 68.5, df = 33, *p* = 0.009, Fig. 2A,C). Similar results were obtained using mixed linear model over single trials (interaction between trial number and group: t_1628_ = −2.68, *p* = 0.0073). Using the same analysis described for accuracy above, we observed that control subjects trend to decrease their RTs as the congruent block elapses (rho = −0.62, df = 23, *p* = 0.002). In contrast, ADHD subjects do not show this trend (rho = 0.15, df = 23, *p* = 0.5, difference z = 3.22, df = 33, *p* = 0.0013, mean rho per subject ADHD: −0.02, df = 16, *p* = 0.5, TD: −0.11, df = 16, *p* < 0.001, diff Wilcoxon test, w = 212, df = 33, *p* = 0.019, Fig. 2). Similar results were obtained using a mixed lineal model over single trails (interaction between trial number and group: t_1628_ = −2.9, *p* = 0.0035, Fig. 2B,D).

### Neuroimaging Results

**Figure 3:**
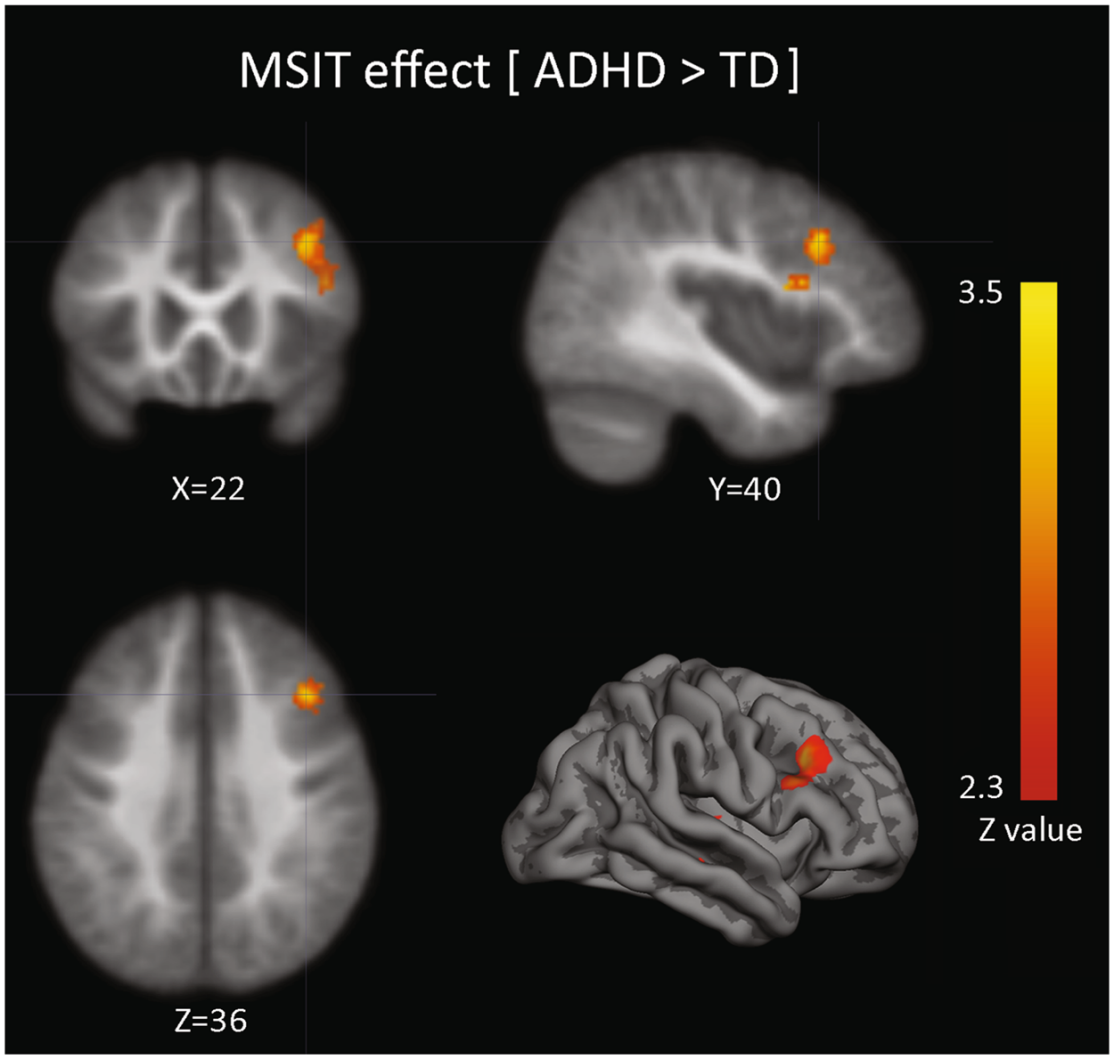
Group differences elicited by MSIT-effect (ADHD[InC-CC] > TD[InC-CC] children).

In order to determine brain activation differences between groups, we studied the MSIT-effect (InC > CC) for ADHD and TD children. For cluster detection threshold, we used a z value of 2.3; and, for cluster correction threshold, p < 0.05. In our task and MRI configuration, this setting has an adequate rate of false positive results (0.049, see Methods and Supplementary Material). Neuroimaging results showed a greater activation of the right medial and inferior frontal gyrus for the ADHD group (Fig. 3). Peaks of activity for this cluster are shown in Table 2.

**Table 2:** Peaks of activity of Group differences (ADHD > TD children) elicited by MSIT-effect (InC > CC).

Areas	X	Y	Z	Z-value	Cluster size	P corrected
	56	18	24	3.44	1366	0.043
	42	16	20	3.41		
	40	22	36	3.29		
	44	4	12	3.18		
	40	10	18	3.09		
	56	6	42	3.08		

**Figure 4:**
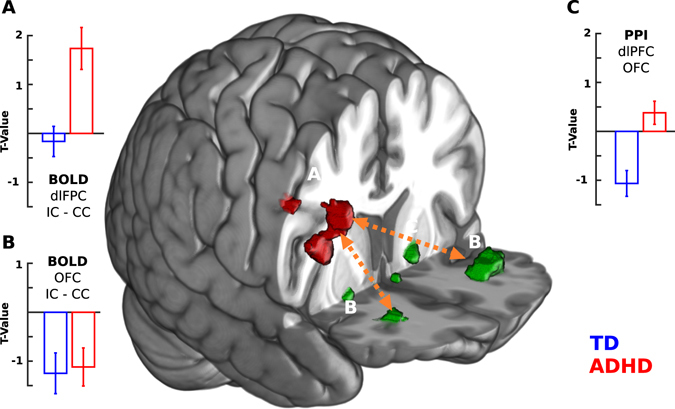
Contextual increases in functional connectivity of rlPFC with bilateral OFC. (**A**) BOLD signal for the MSIT-effect of the cluster depicted by the red volume of interest (VOI) in the 3D brain representation. (**B**) BOLD signal (MSIT-effect) of the significant clusters obtained by PPI analysis (depicted by the green VOIs in the 3D brain representation, using a Z threshold of 3 for visualization). (**C**) PPI representation of the connectivity between (**A,B**)epicted by the orange dotted arrows (Note that the red volume is only for illustrative purpose. See Methods for details). (**A**–**C**) Red bars represent ADHD group and blue bars TD group.

In order to elucidate the functional connectivity of the rlPFC (right middle and inferior frontal gyrus; rMFG, rIFG), a psychophysiological interaction (PPI) analysis was performed using the main cluster of the ADHD > TD MSIT-effect as seed. To avoid circular inference, per each subject we used the result of the contrast excluding the subject’s image (see methods for more details). This analysis showed that the ADHD group presented a significantly greater functional connectivity with bilateral orbitofrontal cortex (OFC) and striatum as compared to the TD group (Mixed effect model, p < 0.01 corr.) (Fig. 4, Table 3).

**Table 3:** Coordinates and statistics of the functional connectivity clusters determined by PPI.

Areas	X	Y	Z	Z-value	Cluster size	P corrected
Left Putamen	−18	8	−2	3.5	**2523**	**0.0017**
Right Middle Frontal gyrus	26	38	8	3.2		
Left Insular Cortex	−30	12	−12	3.1		
Left OFC	−14	12	−14	3		
Right Ventral Striatum	20	10	−8	3		
Left OFC	−30	31	12	2.9		

A correlation analysis between performance during the incongruent condition and PPI connectivity between rlPFC (rMFG and rIFG) and OFC showed that the connectivity between these brain regions is positively correlated with a worse performance in both groups (Table 4). Additionally we explored the correlation between performance during incongruent condition and connectivity between rlPFC and VS, and we did not find significant results (Table 5).

**Table 4:** Correlation between performance during incongruent condition and connectivity between rlPFC and OFC for all subjects.

	Canonical regression			Robust regression		
	slope	t-vale	p-val	slope	t-vale	p-val
rlPFC – OFC r	0.031	1.07	0.29	0.032	1.02	0.31
rlPFC – OFC l	−0.06	−2.2	**0.0348**	−0.06	−2.07	**0.046**

**Table 5:** Correlation between performance during incongruent condition and connectivity between rlPFC and VS for all subjects.

	Canonical regression			Robust regression		
	slope	t-vale	p-val	slope	t-vale	p-val
rlPFC – VS r	0.0325	1.2846	0.2085	0.0333	1.2481	0.2213
rlPFC – VS l	−0.0212	−0.9062	0.3718	−0.0220	−0.8928	0.3789

## Discussion

The main objective of this article has been to assess interference control processing in ADHD children. We found that ADHD children deviate from typically developing children both in the behavioral and functional domains. Performance differences between ADHD and TD children have been widely reported using different methodologies and cognitive tasks^36^. In spite of large studies and meta-analyses of behavioral studies discussing the relative importance of executive function as a key marker for diagnosing ADHD^37–39^, inhibition impairment is consistently found in ADHD children. In fact, evidence of impoverished inhibition skills in ADHD comes from studies that have used motor inhibition tasks^20, 40–42^, delayed response tasks and interference tasks^21, 43, 44^ among others. Poor behavioral inhibition is also evident in deficient performances in tasks that demand the detection of ongoing modifications of rules and the implementation of different strategies in order to respond properly, like the Wisconsin Card Sorting Test^45^. Although current evidence remains controversial, it suggests that ADHD children have problems of response perseveration in a similar manner as those of patients with frontal lobe damage^42, 46^. In accordance, we found that in the first five trials of each block, TD children increased their accuracy in a steeper slope than ADHD children (Fig. 2). Thus, the inability to adequately modify ongoing strategies that are necessary to solve the task properly can be interpreted in terms of the delayed-emergent frontal functioning^22^.

Current evidence shows that ADHD children present a delay in cortical development^13, 16^, which is more prominent in prefrontal association cortex and in the right hemisphere as shown by structural studies^18, 47^. This information is relevant in light of the fact that many of the symptoms of ADHD decrease with development, specifically towards the end of adolescence and adulthood, when frontal cortex is settled and the frontal regions become myelinated^48^. Nevertheless, little is known about functional maturation in ADHD. Current evidence indicates that during typical development, prefrontal activity related to interference control presents a specific pattern of maturation^22^. On one hand, the activity in the dorsal part of the anterior cingulate cortex (dACC) increases with age, evidencing a more prominent process of monitoring performance^25, 49^. On the other hand, the activity in dlPFC related to inhibitory control seems to decrease with age^25, 50^. This change can be interpreted as a reduction in effort whereas a more efficient cortical processing is carried out in order to solve the task^22^. Interestingly, the MSIT produces reliable and robust activation of both dACC and dlPFC as well as the rest of the cingulo-fronto-parietal attention network^31^. Although our results did not directly measure developmental alterations, the use of this task gives the opportunity to study the different components of the frontal circuit related to interference control.

When we compared the brain activation patterns elicited by the MSIT-effect between ADHD and TD groups, differences were significant only for the ADHD > TD contrast (Fig. 3). These differences were placed in a cluster with the activity peak located in the rlPFC. Thus, this over-activation could be interpreted in terms of a cortical compensatory mechanism in order to maintain an adequate performance during the task. Previous works have shown how cortical compensatory activity is a natural and relievable brain mechanism to cope with deficits in several cognitive processes, like response inhibition in ADHD^35, 51–53^, diffuse axonal injury^54^, multiple sclerosis^55, 56^ and schizophrenia^57^. The structural maturation delay of the dlPFC has also been related to persistent inattention symptoms in adults^18^. Thus, this functional alteration could be a marker of symptoms in adults, and can be related to other problems such as social anxiety disorder^4^.

Interestingly, frontal structures have been related to flexible behavioral change and task switching^58–60^. Dorsolateral prefrontal cortex encodes different task rules and participates in behavioral change when the environment requires to change a strategy^61–63^. It has also been shown that the connectivity with ventromedial prefrontal cortex and OFC is important to implement flexible changes^64^. Thus, these areas participate in integrating different information in order to flexibly adapt our behaviors^65–67^. For example, dlPFC -as part of the frontoparietal control network - changes its connectivity with other brain networks depending on the requirements of a specific task^68, 69^. In our contextual functional connectivity analysis we found that ADHD children present more connectivity between rlPFC and OFC during MSIT-effect than TD children. This result is mainly due to a modulation of the connectivity in TD children (see Fig. 4C, negative t-value indicates more connectivity in CC). Studies on healthy children have found that more accurate responses in MSIT are related to more modulation between task positive and task negative networks^24^. In accordance with this, we found that increasing contextual connectivity between rlPFC and bilateral OFC correlated with worse performance. This can be interpreted in the sense that subjects with more connectivity changes between conditions present a better performance. Resting state connectivity studies have shown that ADHD subjects present a less conspicuous anti-correlation between task positivity and task negative networks. Indeed, it has been proposed that immaturity of task positive networks (saliency and fronto-parietal networks) are related to inattention and response variability in ADHD children^70–72^. Interestingly, prior work in adults with ADHD shows alterations in the OFC activation during reward task and risky decision making^29, 73^. Thus, the altered pattern of OFC connectivity can also be related with a more impulsive response^29^.

Finally, taken together these results show that ADHD children have a deficit in interference control that is partly compensated with higher activity in the rlPFC. This activity seems to be modulated by contextual functional connectivity with OFC. In the same line, the recruitment of prefrontal resources as part of a compensation mechanism behavior could reduce the availability of such higher-order prefrontal resources to solve other tasks. It should be considered that these compensatory brain activations involve an additional metabolic cost, which could lead to shorter periods of focused attention, reduced ability to inhibit stimuli interfering, longer reaction times and a generally impoverished behavioral performance. Despite the fact that ADHD children who participate in our experiment did not take medication 24 hours previous the MRI session, it is impossible to rule out an effect of medication^74^. Thus, it is important to carry out similar experiments in pharmacologically naïve patients or their relatives to weigh the relative influence of medication on these findings.

The use of neuroimaging techniques in conjunction with paradigms from neuropsychology, highlight the importance of the complementary use of these approaches^75^, because by themselves behavioral studies are blind to possible underlying brain compensatory mechanisms that, in our study offer an explanation of why the ADHD group maintains an overall similar behavioral performance to the TD group.

## Methods

### Participants

Forty male children aged 10 to 12 years, 20 ADHD-combined type and 20 age and IQ-matched typically developing children, participated in the study (Table 1). The ADHD group participants met DSM-IV criteria for the ADHD combined sub-type and had no major comorbidity. They were recruited from general (secondary care) psychiatric and neurological outpatient services. All of them had a clinically proven history of good response to stimulant medication. All children in the ADHD group were treated with Methylphenidate during at least 3 years before the study (mean 4.8, std.dev. 1.9 years). They were asked to interrupt stimulant treatment the day the exam was taken. All participants were scanned between 10 a.m. and 12 p.m. on Saturdays. The TD group was selected out of a large group who volunteered for the study from public schools. They underwent a complete physical and psychological examination and were classified using the same instruments as the ADHD group: Conners’ Abbreviated Parent-Teacher Questionnaire^76^ and DSM-IV. All participants were Chilean, native Spanish speakers, had normal or corrected to normal vision, no color-vision deficiency and were right handed according to the Edinburgh Inventory^77^. They had an average or higher IQ assessed by WISC-R^78^, and agreed to be examined to rule out any morbidity^79^.

All experimental protocols were approved by the ethics committee of the Clínica Alemana de Santiago de Chile. Written informed consent was obtained from all children and their parents after detailed explanation of the scope of the study. All experiments were performed at the Departamento de Imágenes, Clínica Alemana de Santiago in accordance with the approved guidelines.

### Experimental paradigm

This study applied the cognitive paradigm “Multi Source Interference Task” (MSIT)^31, 80^. This task presents a set of 3 digits on a computer screen. Participants are instructed to report the identity of the number that is different from the other 2 numbers by pressing a button on a control-pad. In the congruent condition, the distracters are zeroes and the target number (1, 2, or 3) is always placed congruently with its position on the pad. In the incongruent condition, the distracters are other numbers (1, 2, or 3), and the target number (1, 2, or 3) is never placed congruently with its position on the response pad (Fig. 1). BOLD signal and behavioral responses were acquired while subjects solved the task. The task was a block design that consisted of five congruent condition blocks alternating with five incongruent condition blocks (total duration: 7 minutes and 27 seconds). Each block consisted of 24 trials, meaning that each subject completed a total of 120 trials per condition. The stimulus sequences were presented on a screen during 860 ms with an inter-stimulus interval of 1000 ms approximately. All participants underwent a training session in a mock scanner that simulated illumination, temperature and sequence noise levels previous to image acquisition, in order to habituate them to the exam conditions.

### Behavioral statistical analysis

Data were tested for normality with the Kolmogorov-Smirnoff test. When data did not meet the normal distribution assumption, nonparametric tests were used. Accuracy and reaction time (RT) were analyzed with mixed ANOVA, using condition (CC or InC) and diagnosis (ADHD or control) as factors. Differences of RT for MSIT-effect (individual contrast of RT in InC > CC) between groups were analyzed with the Wilcoxon test. Changes of the RT for MSIT-effect during congruent blocks were analyzed with Spearman correlation analysis, and differences between groups were analyzed with Wilcoxon signed-rank test. Finally, mixed linear model over single-trial was used to complement the Spearman correlation analysis, using trial number, group and the interaction between them as regressors. Errors were clustered using subject as a grouping factor.

### Image acquisition

Images were acquired at the Department of Radiology and TAC of the Clínica Alemana de Santiago, in a GE HDX 1.5 T MRI gradient 33 mT/M scanner. A volumetric 3D sagittal T1-weighted SPGR sequence was used for acquiring structural data, with TE 4.7, TR 14.5 ms, TI 500 ms, FOV 25 cm, matrix size 320 × 256, flip angle 12°, slice thickness 1.2 mm, 166 slices per volume, NEX 1, 63.84 KHz bandwidth, 6.5 min total time. A T2-weighted sequence was used for acquiring functional data, with 43 slices per volume (covering the whole brain), TR 2516 ms, TE 35 ms, FOV 24 cm, flip angle 90°, matrix size 64 × 64, slice thickness 3 mm, 63.8 KHz bandwidth, Ten blocks of 24 trials each one (240 trials in total) were presented to the subjects within 7 minutes and 27 seconds on the screen. The block duration for both conditions was 44.7 seconds, and then the General Linear Model was fitted using these values. Data sets from 34 of our 40 participants met criteria for high quality and scan stability with minimum motion correction (<3 mm displacement in any direction) and were subsequently included in the fMRI analysis.

### Image processing and analysis

Functional and structural imaging data were preprocessed and analyzed using the FMRIB Software Library (FSL, version 5.08, http://www.fmrib.ox.ac.uk/fsl/index.html). The first five volumes from the time series of each run were discarded to allow the hemodynamic response to stabilize. Data preprocessing involved the following steps: motion correction (MCFLIRT), brain extraction (BET), spatial smoothing with a 8-mm FWHM Gaussian kernel, and high pass temporal filtering using Gaussian-weighted least-squares straight line fitting with sigma = 100.0 s, and pre-whitening. The BOLD response was modeled using a separate explanatory variable (EV) for each task condition (congruent and incongruent). Then the block design was convolved with a double gamma function to produce an expected BOLD response. The temporal derivative of this time-course was then included in the model for each EV to capture any unexpected temporal shifting, and motion correction parameters were also included in the design as additional nuisance regressors. Data for each condition were fitted to the general linear model. Estimated beta maps for contrasts were normalized to MNI152 standard space for each participant for subsequent group comparisons using FLIRT in two stages. First, functional images were aligned with the high-resolution T1 using a six-degrees-of- freedom rigid-body warp. Then, the T1 was registered to the standard MNI atlas with a 12-degrees-of-freedom affine transformation. Second-level activation maps were calculated with FSL using mixed-effect model (FLAME1 + 2).

We also conducted psycho-physiological interaction (PPI) analyses^81^ to investigate functional connectivity of the rlPFC (rMFG, rIFG), which showed significant differences in MSIT-effect (InC > CC) between both groups. The seed was defined per each subject used the following procedure. We calculated the significant cluster of the MSIT-effect (ADHD > TD) using the entire sample excluding the subject’s imaging. Then, the seed was defined as the conjunction between the MSIT-effect cluster and the anatomically defined lateral prefrontal cortex (inferior and middle frontal gyrus, using the Harvard-Oxford Cortical Structural Atlas provided by FSL). Thus, we avoided any circular inference in the results. We then generated a demeaned BOLD time-course regressor from the seed as well as an interaction term with the demeaned psychological regressor (double gamma convolved incongruent conditions time-course) to generate PPI terms. The main effect of the psychological regressor was also included in the GLM. For the PPI time-courses, we subtracted the global mean (the average from all voxels at a specific time point) from the individual time-course of each region investigated, to avoid positive results due to global correlations.

### Statistical thresholding

All results reported were based on an initial uncorrected voxel-level threshold of Z > 2.3 for the cluster detection. Then we corrected the whole brain using the cluster level using p < 0.05. In order to avoid false positive results, al the parameters used in our analysis were tested using the Eklund *et al*., 2016 methodology^82^. The permutation test demonstrated a false positive rate < 0.05 (see supplementary materials).

## Electronic supplementary material

Supplementary Information
