# Supplementary material for: Lateral prefrontal activity as a compensatory strategy for deficits of cortical processing in Attention Deficit Hyperactivity Disorder
Source: Sci Rep. 2017 Aug 3;7:7181. doi: 10.1038/s41598-017-07681-z (PMC5543103; doi:10.1038/s41598-017-07681-z)
Supplement: Supplementary file 1 — Supplementary Information [file 41598_2017_7681_MOESM1_ESM.pdf]

Voxels	P	"-LOG10(P)"	Z-MAX	Z-MAX X (mm)	Z-MAX Y (mm)	Z-MAX Z (mm)	Z-COG X (mm)	Z-COG Y (mm)	Z-COG Z (mm)	COPE-MAX	COPE-MAX X (mm)	COPE-MAX Y (mm)	COPE-MAX Z (mm)	COPE-MEAN
1.96E+03	0.0239	1.62	3.46	38	-6	20	43.5	-21.4	48.3	16.8	34	-34	68	9.57
1670	0.0473	1.33	3.41	10	-68	12	12.4	-76.8	6.31	17.1	6	-88	-10	9.29
3707	0.000918	3.04	3.85	2	-34	56	-0.517	-32	61.9	27.7	-4	-28	76	10.2
1687	0.0471	1.33	3.44	-4	-82	-18	-10.1	-66.4	-19.2	22	-4	-92	-20	8.56
1813	0.0315	1.5	3.77	4	-44	-14	4.47	-32.6	-16.5	16.4	18	-28	-22	8.51
1.32E+04	6.29E-10	9.2	3.76	46	-26	-10	17.9	9.18	10.6	26.2	0	58	26	9.12
7583	2.68E-06	5.57	3.87	18	-32	16	-1.93	-49.3	4.84	21	48	-66	38	8.76
1829	0.0335	1.48	3.46	64	-20	-2	39.4	-14.8	-6.43	14.7	64	-22	-6	7.78
1976	0.0243	1.61	3.67	-28	40	44	-20.9	42.8	34.2	17.4	-8	68	14	10.1
1.65E+04	3.92E-11	10.4	3.8	48	24	-12	18.5	-18.2	29.7	32.4	56	16	-4	10.3
27299	7.22E-16	15.1	4.09	-24	-52	70	7.86	-21.5	30.9	27	-4	-18	74	10.2
2424	0.00981	2.01	3.73	20	2	50	11.8	-17.2	52.8	16.3	40	-32	64	7.81
3155	0.0021	2.68	3.53	22	-64	38	9.48	-72.6	34	30	8	-80	48	12.2
4013	0.0004	3.4	3.7	-42	-28	26	-37.4	-50.1	-0.618	24.8	-52	-64	-24	7.87
1.78E+04	9.03E-12	11	4.11	6	-64	46	1.89	-53.7	18.5	34.9	-2	-56	64	10.6
9.20E+03	2.38E-07	6.62	3.79	26	36	8	10.6	13.9	20.6	28.4	0	-40	76	9.61
2111	0.0178	1.75	3.37	66	-52	32	55.7	-48.7	6.78	19.9	54	-64	26	10.4
4.16E+03	0.000318	3.5	3.53	40	4	-10	15.2	-15.4	5.09	16.4	62	-8	8	7.67
2.60E+03	0.00652	2.19	3.7	2	-66	60	-5.08	-17.2	34.1	31	2	-64	60	9.32
3.71E+03	0.000799	3.1	3.66	24	18	18	-3.07	22.8	10.2	11.6	2	28	6	5.4
8614	7.75E-07	6.11	3.91	-26	-44	-2	-11.1	-51.5	5.78	20.1	2	-44	4	9.32
3451	0.00145	2.84	3.88	-10	-66	56	-6.83	-51.4	45.8	31.5	-2	-66	56	11.9
2199	0.0157	1.8	3.81	16	-22	32	5.32	-14.5	31.5	11.7	2	0	34	5.64
38362	1.48E-20	19.8	3.79	38	-72	50	-6.34	-19.3	21.9	40.1	0	-50	68	10.2
28583	4.15E-16	15.4	3.91	8	-10	32	-5.16	-18.2	22.8	36.3	2	-42	74	9.68
1711	0.0404	1.39	3.79	36	-82	-16	9.99	-68.4	-12.3	16.2	10	-88	-16	8.85
3152	0.00212	2.67	3.59	28	-16	50	10.9	-36.3	21.4	18.7	0	-56	16	8.73
9921	5.96E-08	7.22	3.51	30	-46	-24	4	-56.4	-8.04	25.5	40	-80	-24	9.17
7363	3.52E-06	5.45	3.73	4	-32	40	-9.8	-29.4	23.7	18.9	0	-46	6	7.08
4032	0.000488	3.31	3.95	-8	32	-10	-4.18	32.5	1.4	20	0	26	-12	6.13
1853	0.0315	1.5	3.41	14	-84	8	4.5	-80.4	11.4	14.8	10	-82	8	8.28
2007	0.0219	1.66	3.53	54	-66	-30	26.9	-81.8	-19.1	31.3	22	-90	-20	15.5
4021	0.000456	3.34	3.8	-2	-18	70	2.99	-2.35	52.1	39.8	0	-26	74	13.1
46038	3.16E-22	21.5	4.54	8	-78	34	-3.27	-17.4	14	32	0	-66	70	9.73
2825	0.00414	2.38	3.47	8	-32	6	4.33	-31.5	14.1	21.7	2	-70	46	8.4
3004	0.003	2.52	3.48	4	-6	4	0.581	-17	13.6	16.7	0	-46	2	8.24
3061	0.00285	2.55	3.39	-46	-72	-6	-44.2	-37.2	-4.31	17.3	-52	-70	-10	7.82
12301	4.21E-09	8.38	3.64	-26	44	24	-0.0095	13.3	32.5	30.4	0	-26	72	9.73
2112	0.0169	1.77	3.48	-44	-72	-8	-37.5	-60.6	0.759	17.3	-52	-72	-8	7.09
2850	0.00411	2.39	3.81	6	-26	54	21.5	-25.8	59.7	20.1	40	-30	64	10.4
1861	0.0311	1.51	3.49	8	-38	32	6.41	-48.9	26.6	15.8	4	-68	30	9.09
3150	0.00248	2.61	3.62	48	-10	2	35.9	4.88	2.88	20.8	54	12	-2	8.2
27415	4.06E-16	15.4	4.06	10	-58	24	-12.7	-52.4	20.5	28.5	2	-90	42	10.5
13589	7.83E-10	9.11	4.02	-14	8	-14	-4.39	-28	10.1	32.4	2	-64	60	9.5
1875	0.0274	1.56	3.31	-8	-100	12	-20.5	-90.1	7.45	17.5	-2	-96	14	9.28
13578	5.12E-10	9.29	3.99	24	-82	22	3.6	-76.5	-2.39	27.6	2	-96	14	10.6
12674	2.52E-09	8.6	3.8	48	-50	44	1.43	-39.8	16	32.2	0	-42	74	9.92
8411	7.15E-07	6.15	3.86	44	-78	-8	34.2	-59.4	5.86	24.4	40	-80	-18	9.53
1705	0.0397	1.4	3.49	-2	-72	42	-2.85	-55.7	38.9	26	-2	-56	62	12.7
